# EDM-Net: A Multi-Scale Network for Object Detection in Remote Sensing Images

**DOI:** 10.3390/s26123927

**Published:** 2026-06-20

**Authors:** Shuai Liang, Xiao Wang, Jialong Sun, Hui Liu, Huilei Yang

**Affiliations:** 1 School of Marine Technology and Geomatics, Jiangsu Ocean University, Lianyungang 222000, China; 2 Key Laboratory of Marine Meteorological Disaster Prevention and Mitigation of Jiang Su Province, Lianyungang 222005, China

**Keywords:** remote sensing object detection, intra-scale feature interaction, cross-scale feature fusion, transformer, multi-scale object detection

## Abstract

Remote sensing object detection remains challenging because objects often appear with large scale variation, dense spatial layouts, and strong interference from complex geographical backgrounds. To address these coupled difficulties, we propose EDM-Net, an end-to-end multi-scale detector that organizes feature processing into three coordinated stages: adaptive extraction, intra-scale interaction, and cross-scale fusion. First, an efficient sparse mixture-of-experts (ES-MoE) module is embedded in the backbone to allocate scale-specific convolutional experts according to scene-level feature responses, providing a more adaptive feature basis than a single static extraction path. Second, a dynamic mixing intra-scale feature interaction (DMIFI) module is introduced into the Transformer encoder. This module combines global self-attention with dynamic spatial mixing, thereby preserving long-range context while reintroducing local two-dimensional inductive bias for dense and small objects. Third, a multi-scale synergistic attention fusion (MSAF) module aligns adjacent feature levels through parallel local and global attention branches and structural re-parameterization, reducing semantic dilution during feature aggregation. Comprehensive experiments on three large-scale remote sensing benchmark datasets, DIOR, NWPU VHR-10, and RSOD, demonstrate that EDM-Net consistently improves over the re-trained RT-DETR-R18 baseline under the same experimental protocol, attaining mAP50 scores of 83.7%, 95.6%, and 95.8% respectively. Additional ablation and scale-specific analyses indicate that the three modules contribute complementary gains, especially for small and densely distributed objects. These results suggest that coordinated extraction, interaction, and fusion can improve remote sensing object detection under complex scale and background conditions.

## 1. Introduction

**Figure 1:**
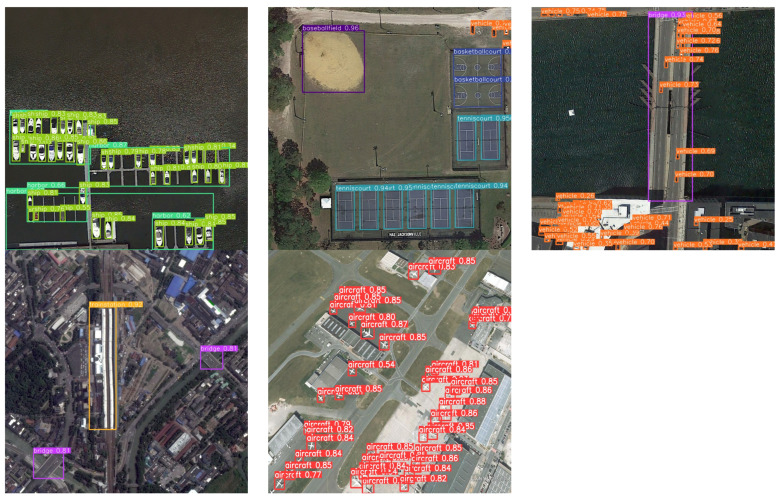
Objects in remote sensing images exhibit significant scale variations.

The increasing availability of high-resolution optical remote sensing images provides extensive application in several fields such as land use and land cover mapping, urban planning, and environmental monitoring [1,2,3]. The accurate identification and localization of targets of interest within vast amounts of remote sensing data have become essential for interpretation and analysis of large-scale geospatial imagery, drawing widespread attention in the interdisciplinary fields of computer vision and earth sciences [4,5,6]. However, due to the differences in sensor parameters, platform heights, and observation angles [7,8,9]. Remote sensing images may exhibit considerable scale variations within the same scene [10,11,12,13]. As shown in Figure 1, large landmark buildings and miniature vehicles may coexist in one image. This scale diversity poses challenges to detection algorithms. Furthermore, due to complex land cover types and variable lighting and atmospheric conditions, objects are often submerged in heavily textured backgrounds [14]. Directly applying general detectors developed for natural scenes struggles to achieve ideal results [15]. Therefore, there is an urgent need to design detection algorithms specifically tailored to the characteristics of remote sensing images.

Early research on remote sensing object detection mostly relied on handcrafted features, such as shape and texture information [16], saliency features [17], and scale-invariant features [18]. These methods performed reasonably well under controlled conditions, but their generalization ability and descriptive capacity were insufficient when facing significant variations in object appearance. Convolutional neural network (CNN)-based detectors, such as the R-CNN series [19,20,21], YOLO series [22,23,24,25,26,27], and SSD series [28,29], significantly improved detection accuracy. However, the local receptive fields of CNNs limit their ability to capture long-range dependencies. When dealing with extremely small or densely distributed objects, the feature representation is inadequate and susceptible to interference from complex background noise. Moreover, such detectors typically rely on handcrafted components like non-maximum suppression (NMS), which not only introduce additional computational overhead but also constrain the overall detection performance to some extent. These limitations make it difficult for models to precisely focus limited representational capacity on critical regions, inevitably leading to missed and false detections.

The introduction of the Transformer architecture to computer vision has provided a new paradigm for object detection. DETR [30] discards anchor boxes and non-maximum suppression (NMS), reformulating the detection task as a set prediction problem and achieving true end-to-end detection. Subsequent works such as Deformable-DETR [31] focus on sparse keypoint sampling via a deformable attention mechanism, improving computational efficiency; DINO [32] introduces a contrastive denoising strategy to enhance the discriminative ability of queries. Despite their impressive performance in natural scenes, existing DETR variants still exhibit inadequate adaptability when applied to remote sensing images.

Crucially, most existing works attempt to address remote sensing challenges by introducing isolated improvements, neglecting the necessity of a highly coupled, synergistic system. Specifically, the standard backbone network applies a unified static computation path to all spatial positions, failing to adaptively allocate resources according to scene complexity. Meanwhile, the standard Transformer encoder is confined to global attention and lacks the capability to perceive two-dimensional local spatial structures, which tends to cause excessive smoothing of object boundaries in complex backgrounds. Finally, existing multi-scale fusion strategies neglect the semantic gap between deep semantic and shallow detail features, causing the discriminative information of small objects to be easily diluted during the fusion process.

To address these coupled limitations, we propose EDM-Net, a multi-scale remote sensing detector that explicitly links feature extraction, intra-scale interaction, and cross-scale fusion. The central design principle is not to add isolated enhancement blocks, but to preserve scale-sensitive information through the entire detection pipeline. ES-MoE first provides adaptive multi-receptive-field extraction in the backbone. DMIFI then refines the deepest feature level by combining global self-attention with dynamic two-dimensional spatial mixing. MSAF finally aligns adjacent feature levels before fusion so that low-level spatial details and high-level semantics are combined with less semantic dilution.

The main contributions are summarized as follows.

1. We propose EDM-Net, an end-to-end multi-scale detection framework for optical remote sensing images. The framework explicitly decomposes the detection pipeline into adaptive feature extraction, intra-scale feature interaction, and cross-scale feature fusion, providing a structured way to address scale variation and background interference.
2. We introduce ES-MoE into the backbone to replace a single static extraction path with sparsely activated depthwise convolutional experts. Different expert branches use different kernel sizes, allowing the backbone to adjust its effective receptive field according to scene-level feature responses.
3. We design DMIFI for the Transformer encoder. DMIFI restores the two-dimensional feature layout after global attention and applies dynamic spatial mixing, which strengthens local spatial bias for dense small objects.
4. We develop MSAF for multi-scale feature aggregation. The module combines channel projection, local–global attention branches, and structural re-parameterization to reduce the semantic gap between adjacent feature levels while maintaining an efficient inference form.
5. We validate EDM-Net on DIOR [33], NWPU VHR-10 [34], and RSOD [35]. In addition to overall mAP50 comparisons, we report ablation studies and scale-specific AP analyses to examine the contribution of each module and the behavior of the model on small, medium, and large objects.

## 2. Related Work

### 2.1. Remote Sensing Small Object Detection

Small object detection has always been one of the most challenging problems in the field of remote sensing image analysis. In remote sensing images, small objects are typically defined as instances with a pixel area smaller than 32 × 32, following the widely adopted MS COCO evaluation metrics and standard conventions in aerial image analysis [36,37]. The absolute scale of the target is tiny, occupying only a very limited pixel region in the entire image, and the discriminative information carried by the object itself is naturally sparse. In addition, multiple downsampling operations in the backbone network further compress the already cramped target region in the feature maps, making small objects easily smoothed or even submerged in deep features. According to statistics from large-scale datasets such as DIOR [33], such small objects account for more than half of the total, making this problem of universal research significance and urgent application demand. To address the above challenges, researchers have carried out active explorations. Wu et al. [38] introduced the classic SE channel attention module into the backbone network to enhance the model’s attention weight to shallow-level small object features. Han et al. [39] proposed CSADet, which utilizes contextual information surrounding the target to assist in learning small object features. Deng et al. [40] constructed the Extended Feature Pyramid Network (EFPN), which preserves richer spatial structure information for small objects by introducing an additional high-resolution pyramid level.

Although traditional methods enhance object visibility, they fail to separate fine textures from complex backgrounds, leaving boundaries blurred. To address this, we propose a cascaded sharpening approach rather than simple attention scaling, fundamentally restoring the structures of small objects.

### 2.2. Multi-Scale Feature Extraction and Fusion

To cope with the dual challenges of extreme scale variation and complex background interference in remote sensing images, multi-scale feature processing has become a key technical path for improving detection performance, and research is mainly carried out along feature extraction and feature fusion.

In terms of multi-scale feature extraction, researchers are committed to enabling the network to capture target representations at different scales. Gao et al. [41] designed the Partial and Pointwise Convolution Extraction Module (P^2^CEM), which extracts information of different granularities in parallel through spatial and channel dual branches. Zhang et al. [42] constructed a lightweight feature pyramid, using deformable convolution and Pixel-Shuffle to dynamically adjust the receptive field and reorganize channel features. Zhang et al. [43] proposed a scale-adaptive module, expanding the receptive field range through a combination of standard convolution and dilated convolution. Shi et al. [44] designed the Multi-scale Feature Extraction Module (MFEM), which uses multiple parallel pooling layers to obtain features of different receptive fields at extremely low computational cost. Wu et al. [45] proposed the Local–Global Spatial Feature Enhancement (LGSFE) module, which simultaneously captures local details and global semantics through a receptive field attention mechanism. These methods have made progress on specific scale issues, but their core still relies on a static computation paradigm, which cannot dynamically adjust resource allocation according to the spatial complexity of the remote sensing scene, and thus has an inherent adaptation defect when facing scenes with extremely large scale spans.

Multi-scale feature fusion and context modeling are devoted to bridging the semantic gap between shallow features and deep features. Feature Pyramid Networks (FPNs) [46] transmit high-level semantic information through a top-down path, enabling the network to simultaneously capture fine-grained details of small objects and global semantics of large objects. Subsequent improvements have advanced this direction from different angles: Ghiasi et al. [47] introduced NAS-FPN, which introduces architecture search to automatically design fusion structures. Tan et al. [48] proposed BiFPN, using a weighted bidirectional network to achieve efficient cross-scale interaction. Liu et al. [49] designed PAFPN, which supplements shallow detail information through a bottom-up path enhancement. In addition to pyramid structures, context information modeling is another effective path. By mining the associations between a target and the surrounding environment or other targets, context information can effectively enhance target recognition capability. Zhang et al. [50] constructed the ForDet framework, which highlights potential foreground anchors by injecting foreground contextual representations, effectively enhancing the detection performance of small objects. In terms of context region utilization, Zhang et al. [10] simultaneously captured scene-level and object-level context information and introduced a spatial scale-aware attention module to solve detection challenges such as sparse texture and low contrast.

These studies show that multi-scale extraction and feature fusion are both essential for remote sensing detection. However, most existing methods optimize one stage of the pipeline at a time. Static multi-branch extraction improves receptive field diversity but does not explicitly select computation according to scene complexity. Pyramid fusion methods improve information flow across levels, but they may still mix shallow details and deep semantics without sufficient alignment. EDM-Net is designed to address these two issues jointly. ES-MoE introduces sparse expert selection during feature extraction, whereas MSAF performs attention-based alignment before cross-scale aggregation. This design distinguishes EDM-Net from methods that only enlarge receptive fields or only redesign the fusion topology.

### 2.3. Transformer-Based End-to-End Object Detection

Driven by this demand, the Transformer architecture, empowered by the powerful global dependency modeling capability of the self-attention mechanism, has fundamentally changed the computer vision paradigm. DETR, proposed by Carion et al. [30], redefines detection as set prediction and achieves end-to-end detection through an encoder–decoder structure, discarding traditional components such as anchor box design and NMS. However, DETR converges slowly and has suboptimal small object detection performance, which has spawned a series of improvement works: Deformable-DETR [31] introduces deformable attention, focusing only on sparse sampling points around reference points, significantly reducing computational complexity and supporting multi-scale features. DINO [32] further proposes contrastive denoising training and a mixed query selection strategy on this basis, setting a new performance benchmark. Geared towards real-time application requirements, RT-DETR [51] surpasses the YOLO series in speed for the first time through optimized architecture design, and DEIM [52] reduces training time by half and significantly improves accuracy through dense one-to-one matching and matching-aware loss.

The above general DETR variants have achieved remarkable success in natural scene object detection, but they still face adaptation challenges when directly applied to remote sensing images. The characteristics of remote sensing images, such as large target scale spans, complex backgrounds, and dense distributions of small objects, have prompted researchers to propose a series of specifically improved DETR models recently. Zhao et al. [53] designed Position-DETR, which highlights small target regions and suppresses background interference through hierarchical foreground selection and fine-grained foreground enhancement modules. Zhi et al. [54] proposed SAFF-DETR, starting from the perspective of frequency feature enhancement, constructing multi-branch representation fusion and cross-layer frequency attention mechanisms to improve multi-scale target perception capability. Li et al. [55] built SME-DETR tailored to the characteristics of UAV images, designing a multi-domain enhancement framework to jointly optimize spatial, frequency, and channel domain features, and introduced a background suppression gate to reduce false positives.

Despite their success, existing DETR variants lack 2D local spatial perception, often causing over-smoothed boundaries where dense objects blend into backgrounds [43]. To overcome this, we propose the DMIFI module, which systematically reintroduces local perception by combining global dependencies with dynamic spatial mixing.

## 3. Methodology

### 3.1. Motivation and Overall Architecture

**Figure 2:**
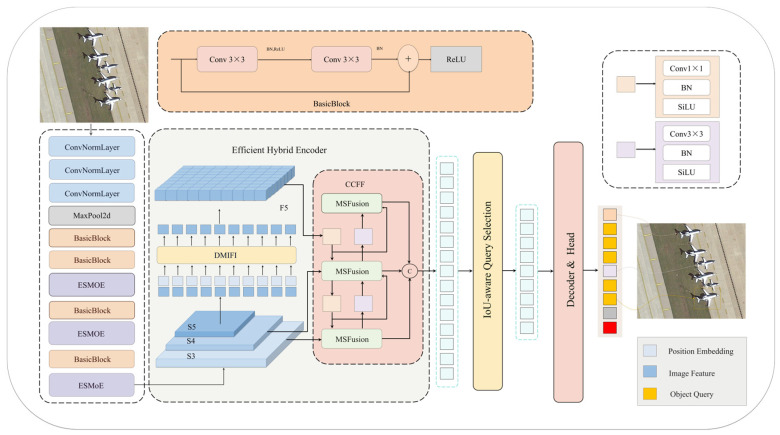
Overall architecture of EDM-Net. Driven by a synergistic extraction–interaction–fusion paradigm, the network sequentially integrates three core modules: ES-MoE in the backbone for scale-adaptive feature extraction, DMIFI in the Transformer encoder for local boundary sharpening, and MSAF in the feature fusion stage for cross-scale semantic alignment.

To overcome the limitations of isolated module designs discussed in Section 2, EDM-Net is formulated as a highly coupled, end-to-end detection system tailored for remote sensing imagery. Rather than simply stacking existing components, our architecture is driven by a synergistic extraction–interaction–fusion paradigm, as shown in Figure 2. EDM-Net is built upon the RT-DETR encoder–decoder detection framework.

Given an input image, the feature extraction process is first governed by the efficient sparse mixture-of-experts (ES-MoE) module [56] embedded in the backbone. This allows the network to dynamically allocate computational resources according to the spatial complexity of the scene, producing scale-adaptive hierarchical feature maps at the source. However, these foundational features alone are insufficient to distinguish dense tiny objects from heavily textured backgrounds. Therefore, the extracted multi-scale features are subsequently fed into the dynamic mixing intra-scale feature interaction (DMIFI) module within the Transformer encoder. By harmonizing global dependency modeling with dynamic spatial mixing, DMIFI systematically re-injects 2D local spatial inductive bias, thereby preventing feature over-smoothing and sharpening object boundaries. Finally, to prevent the semantic dilution of these refined features during multi-scale aggregation, the multi-scale synergistic attention fusion (MSAF) module is employed in the feature fusion stage. Utilizing structural re-parameterization, MSAF ensures deep semantic alignment between high-level context and low-level spatial details.

### 3.2. Efficient Sparse Mixture-of-Experts (ES-MoE)

**Figure 3:**
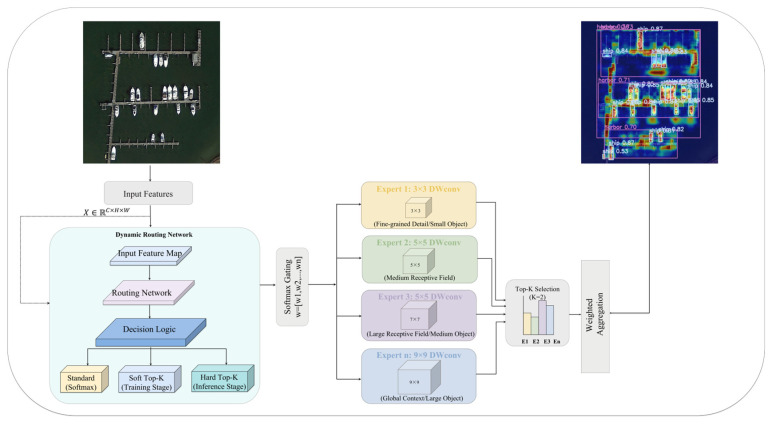
Structure of ES-MoE. This module utilizes a lightweight dynamic routing mechanism to adaptively activate multi-scale experts, allocating computational resources based on input scene complexity to achieve efficient, scale-aware feature extraction.

As the foundational stage of our synergistic architecture, the efficient sparse mixture-of-experts (ES-MoE) module is embedded within the backbone to tackle extreme scale variations at the source. Standard convolutional networks apply a static computational path uniformly across all spatial regions [57]. This rigid paradigm fails to dynamically allocate representational capacity, leading to missed tiny objects and inadequate modeling of large-scale land features. To break this limitation, ES-MoE adopts an instance-adaptive computing paradigm, dynamically activating specialized expert networks based on the local spatial complexity and object distribution of the input remote sensing image, as shown in Figure 3.

To accommodate the drastic object scale variations in remote sensing while adhering to stringent computational constraints, each expert ${\mathrm{Expert}}_{i}$ utilizes a Depthwise Separable Convolution (DWConv) as its fundamental unit rather than standard convolution. Crucially, the DWConv for each expert is configured with differentiated kernel sizes of 3, 5, 7, and 9. This deliberate configuration provides a spectrum of receptive fields—smaller kernels activate for dense, tiny vehicles, while larger kernels capture the macro-context of extensive landmark buildings. The calculation process is shown in Equation (1):

(1) $${\mathrm{Expert}}_{i}\left(X\right)={\mathrm{DWconv}}_{k_{i},{\;C}_{in}\to C_{out}}\left(X\right)$$

To determine which experts to activate, a lightweight gating network G generates instance-aware routing weights based on global scene semantics. Specifically, Global Average Pooling is first applied to the input feature X to extract a global descriptor $P=GAP\left(X\right)$. This descriptor is then processed through two sequential 1 × 1 convolutional layers to produce the raw routing logits $\mathrm{logits}\;\Lambda \in R^{E\times 1\times 1}$. The calculation process is defined in Equation (2):

(2) $$\Lambda ={\mathrm{Conv}}_{1\times 1}^{\mathrm{out}=E}\left(\mathrm{SiLU}\left({\mathrm{Conv}}_{1\times 1}^{\mathrm{out}=C_{\mathrm{red}}}\left(P\right)\right)\right)$$

where the channel reduction ratio γ=8, and $C_{\mathrm{red}}=\max\left(\frac{C}{\gamma },8\right)$. This design strategically decouples the routing complexity from the high spatial resolution of remote sensing images, linking it only to the channel dimension.

To strike an optimal balance between training stability and inference efficiency, ES-MoE employs a dynamic, staged routing strategy. During the training stage, a Soft Top-K mechanism is utilized to ensure continuous gradient flow and promote expert differentiation. During the inference stage, the module seamlessly switches to a Hard Top-K mechanism. Only the outputs of the selected Top-K experts are computed and normalized, while the weights of the remaining experts are set to zero, achieving true computational sparsity without sacrificing accuracy. The calculation process is outlined in Equations (3)–(5).

(3) $$\Omega_{\mathrm{train}}=\frac{\Omega^{\prime }⊙M_{K}}{\sum_{j=1}^{E}\;(\Omega^{\prime }{)}_{j}⊙(M_{K}{)}_{j}+\epsilon }$$

(4) $$\Omega_{\mathrm{infer},i}=\left\{\begin{array}{ll} \frac{exp\left(\Lambda_{i}\right)}{\sum_{j\in I_{K}}\;\exp\left(\Lambda_{j}\right)} & \mathrm{if}\;i\in I_{K}\\ 0 & \mathrm{otherwise} \end{array}\right.$$

(5) $$\Omega =\left\{\begin{array}{ll} \Omega_{\mathrm{train}} & \mathrm{if}\;\mathrm{Training}\\ \Omega_{\mathrm{infer}} & \mathrm{if}\;\mathrm{Inference} \end{array}\right.$$

By performing this on-demand scale allocation at the source, ES-MoE effectively purifies the multi-scale representations. These scale-adaptive features are then seamlessly passed to the subsequent DMIFI module, which further refines their local spatial boundaries against complex backgrounds.

### 3.3. Dynamic Mixing Intra-Scale Feature Interaction (DMIFI)

After extracting scale-adaptive hierarchical features via the ES-MoE module, the deepest layer of the backbone network, such as the P5 layer, contains extremely rich abstract semantic information, which is critical for the macroscopic recognition of objects. Currently, mainstream end-to-end detectors typically introduce a Transformer encoder at this layer to perform intra-scale feature interaction. However, the Feed-Forward Network (FFN) in a standard Transformer encoder is composed of point-wise Multilayer Perceptrons (MLPs), strictly constrained to 1D sequence processing and lacking the ability to perceive 2D local spatial inductive bias. Given that tiny objects in remote sensing images are often dense and surrounded by complex, unstructured terrain, global interactions lacking spatial inductive bias easily lead to over-smoothing of object boundaries, resulting in increased missed detections.

**Figure 4:**
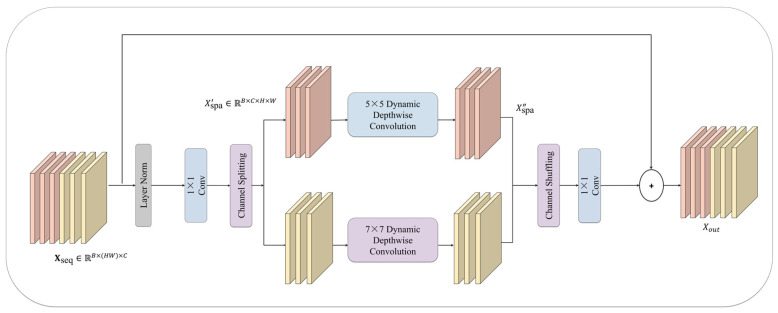
Structure of the DMIFI module. DMIFI first performs global self-attention on the flattened spatial sequence and then reshapes the sequence back to a two-dimensional feature map for dynamic spatial mixing. The self-attention branch models long-range dependencies, whereas the spatial mixing branch enhances local structural representation before cross-scale fusion.

To overcome this bottleneck and bridge the gap between global context and local details, we propose an intra-scale feature interaction module based on a Dynamic Mixing Layer (DML), named DMIFI. Functioning as the intermediate interaction stage of our synergistic pipeline, DMIFI re-injects adaptive 2D local spatial priors while preserving the Transformer’s superiority in global dependency modeling. As shown in Figure 4, its processing pipeline seamlessly integrates two stages: global semantic interaction and 2D dynamic spatial mixing.

First, given the 2D spatial feature $X\in R^{B\times C\times H\times W}$ output by the deep layer of the ES-MoE equipped backbone, we use a 2D Sin-Cos Positional Embedding $P_{e}$ to explicitly inject spatial coordinate priors, and flatten it into a 1D sequence $X_{\mathrm{seq}}\in R^{B\times \left(HW\right)\times C}$. Subsequently, a Multi-Head Self-Attention (MHSA) mechanism is employed to capture global macroscopic context dependencies. The global interaction process adopting a Pre-Norm structure is shown in Equation (6):

(6) $$X_{\mathrm{seq}}^{\prime }=X_{\mathrm{seq}}+\mathrm{MHSA}\left(\mathrm{LN}\left(X_{\mathrm{seq}}\right)+P_{e},\mathrm{LN}\left(X_{\mathrm{seq}}\right)+P_{e},\mathrm{LN}\left(X_{\mathrm{seq}}\right)\right)$$

where LN(-) represents Layer Normalization. Through this operation, the network can span spatial distances and establish macroscopic topological correlations between large objects and complex backgrounds.

Subsequently, to compensate for the defect of local detail smoothing caused by global interaction, DMIFI discards conventional 1D point-wise feed-forward mapping and innovatively constructs a 2D Dynamic Spatial Mixing mechanism internally to guide the non-linear evolution of features. The critical step involves reshaping the globally interacted sequence $X_{\mathrm{seq}}^{\prime }$ back into the 2D spatial dimension $X_{\mathrm{spa}}^{\prime }\in R^{B\times C\times H\times W}$. Next, this mixing mechanism performs channel splitting on the spatial features and uses parallel multi-scale Dynamic Depthwise Convolutions for local feature reconstruction. Its computational logic is shown in Equation (7):

(7) $$X_{\mathrm{spa}}^{\prime \prime }=\mathrm{Concat}\left(D_{k_{1}}\left(X_{\mathrm{spa},1}^{\prime }\right),D_{k_{2}}\left(X_{\mathrm{spa},2}^{\prime }\right),\ldots \right)$$

where $D_{k_{1}}$ represents dynamic convolution operators with different kernel sizes. Unlike static convolutions, the weights of dynamic convolutions are adaptively generated based on input features, enabling the dynamic adjustment of the receptive field according to diverse geographical textures.

Finally, the spatial features extracted through dynamic spatial mixing are flattened again and subjected to a residual connection with the main stream, completing the intra-scale feature evolution, as illustrated in Equation (8):

(8) $$X_{out}=X_{\mathrm{seq}}^{\prime }+\mathrm{Dropout}\left(\mathrm{Flatten}\left(X_{\mathrm{spa}}^{\prime \prime }\right)\right)$$

From a physical perspective, DMIFI constructs a cascaded perceptual loop moving from global mapping to local sharpening. The MHSA in the first half is responsible for capturing the context relationships of large-scale objects across the entire image scope, whereas the dynamic spatial mixing process in the second half acts like an adaptive spatial magnifying glass, enhancing local boundary representations blurred by global attention through multi-scale dynamic convolutions. By generating these more precise and boundary-aware feature representations, DMIFI adequately prepares the semantic foundation for the final cross-scale alignment in the subsequent MSAF module.

### 3.4. Multi-Scale Synergistic Attention Fusion (MSAF)

Following the adaptive feature extraction by ES-MoE and the intra-scale boundary sharpening by DMIFI, the refined high-level features must be fused with shallow-level details to construct a complete multi-scale representation. However, a significant semantic gap exists between these levels. Traditional Feature Pyramid Networks (FPNs) typically rely on simple isotropic operations, such as element-wise addition or static concatenation. When confronting extreme scale discrepancies, these naive interactions easily lead to the semantic dilution of the sharp discriminative information carefully preserved by the previous modules.

**Figure 5:**
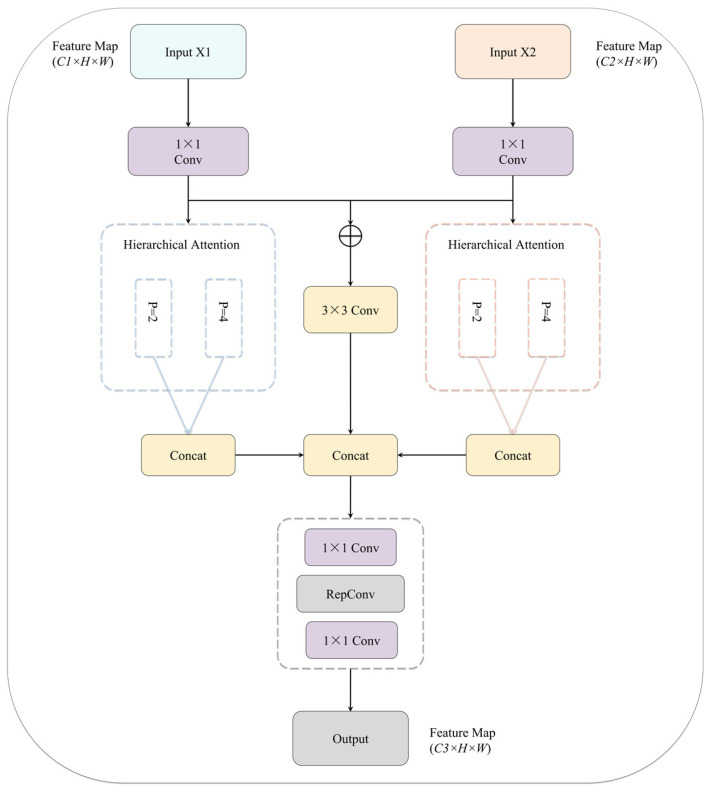
Structure of the MSAF module. As the final stage of the synergistic pipeline, it achieves deep cross-scale feature alignment via parallel local and global attention branches. Utilizing structural re-parameterization, MSAF effectively bridges the semantic gap and prevents the dilution of refined tiny object features without introducing additional inference overhead.

To prevent this semantic degradation and ensure cross-scale synergy, we propose the multi-scale synergistic attention fusion (MSAF) module, as shown in Figure 5. This module aims to achieve precise alignment of cross-level features through parallel multi-granularity perceptual pathways. MSAF’s processing pipeline is divided into three stages: channel projection alignment, dual-path decoupled capture, and re-parameterization aggregation. Given input features $X_{1}$ and $X_{2}$ from adjacent levels, the module first maps them into a unified latent space via projection operators. It then constructs a baseline residual branch $B_{p}$ to preserve original semantics. The computation is shown in Equation (9):

(9) $$B_{p}=F_{G}\left(\phi_{1}\left(X_{1}\right)⊕\phi_{2}\left(X_{2}\right)\right),B_{p}\in R^{C_{\mathrm{out}}\times H\times W}$$

where ϕ represents a 1 × 1 channel adjustment convolution, ⊕ denotes pixel-wise addition, and $F_{G}$ stands for Group Convolution, which is used to establish preliminary cross-level spatial correlations with minimal computational overhead.

Subsequently, to synchronously decouple fine-grained spatial topologies and macroscopic environmental cues from heterogeneous feature streams, MSAF introduces a parallelized Multi-scope Attention Synergy mechanism. For each feature projection $W_{i}$, we simultaneously drive local and global perceptual branches. Through adaptive modulation with different receptive field factors, it achieves object boundary sharpening and unstructured background suppression. Its mathematical expression is shown in Equation (10):

(10) $$Y_{i}=\mathrm{Concat}\left[A_{L}\left(W_{i}\right),A_{G}\left(W_{i}\right)\right],i\in \left\{1,2\right\}$$

where $A_{L}$ focuses on neighboring spatial regions to capture the texture features of small objects, while $A_{G}$ captures macroscopic semantic contexts via expanded receptive fields. This hierarchical attention decoupling design ensures that the network can significantly boost the feature saliency of foreground objects when confronted with complex geographical interference.

Finally, MSAF deeply aggregates the multi-path enhanced feature stream with the baseline residual stream. To optimize edge deployment efficiency while maintaining high accuracy, we incorporate structural re-parameterization convolution (RepConv) for ultimate feature purification. The fusion logic for the output feature $F_{out}$ is shown in Equation (11):

(11) $$F_{out}=\psi \left(\mathrm{RepConv}\left(\sigma \left(\mathrm{Concat}\left(Y_{1},Y_{2},B_{p}\right)\right)\right)\right)$$

where σ denotes the channel compression operation and ψ is the final linear projection. During training, RepConv preserves a multi-branch topology to improve feature transformation. During deployment, the equivalent convolutional kernels and biases are merged into a single convolutional operator. Therefore, the re-parameterized branch does not add extra inference branches after conversion, providing a robust feature foundation for solving the difficulties of dense small object distributions and background aliasing in remote sensing images.

## 4. Experiments

**Figure 6:**
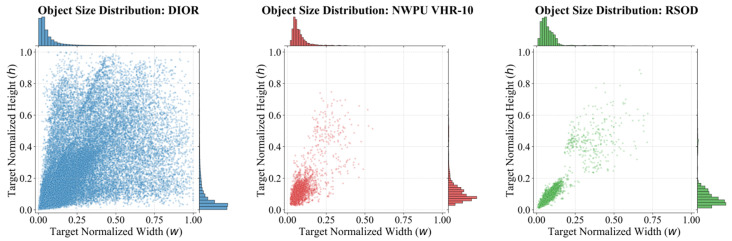
Object size distribution of three remote sensing benchmarks. Closer to the bottom-left corner indicates smaller object sizes, while the top-right indicates larger sizes.

To comprehensively evaluate the detection performance of EDM-Net on remote sensing images with significant target scale variations, we conduct experiments on three large-scale remote sensing object detection benchmark datasets: DIOR, NWPU VHR-10, and RSOD. These three datasets cover a variety of typical detection tasks ranging from high-resolution optical imagery to complex ground object scenes, and their core challenges align closely with the issues this paper focuses on, namely multi-scale variations, complex backgrounds, and dense small object detection. Figure 6 presents the scale distribution of all annotated instances in each dataset in the form of scatter plots, where the horizontal and vertical axes represent the normalized width and height of the target bounding boxes, respectively, and each scatter point represents an independent target instance. The marginal histograms at the top and right further summarize the frequency distribution of targets along the width and height dimensions. It can be clearly observed across all three datasets that the vast majority of scatter points are densely clustered in the small-scale region at the lower-left corner of the coordinate system, and the marginal histograms exhibit a pronounced long-tail distribution pattern—the number of targets peaks at extremely small scales and then declines sharply as the scale increases. This distribution trend indicates that small targets account for an extremely high proportion in remote sensing datasets, with severely imbalanced scale distributions. This is precisely the core difficulty that distinguishes remote sensing object detection from natural scene detection and highlights the scale variation challenges that EDM-Net is designed to address.

### 4.1. Datasets

(1) DIOR Dataset [33]:

This is the largest benchmark dataset for object detection in optical remote sensing images. It contains 23,463 high-resolution optical remote sensing images covering 20 common object categories, totaling 192,512 instances. The image size is uniformly 800 × 800 pixels. In the experiments, categories are denoted as C1–C20: Airplane (C1), Airport (C2), Baseball field (C3), Basketball court (C4), Bridge (C5), Chimney (C6), Dam (C7), Expressway service area (C8), Expressway toll station (C9), Golf course (C10), Ground track field (C11), Harbor (C12), Overpass (C13), Ship (C14), Stadium (C15), Storage tank (C16), Tennis court (C17), Train station (C18), Vehicle (C19), and Windmill (C20).

(2) NWPU VHR-10 Dataset [34]:

This dataset contains 800 high-resolution visible light remote sensing images, covering 3896 object instances across ten categories. The abbreviations for the experimental categories C1–C10 are: Airplane (C1), Ship (C2), Storage tank (C3), Baseball diamond (C4), Tennis court (C5), Basketball court (C6), Ground track field (C7), Harbor (C8), Bridge (C9), and Vehicle (C10).

(3) RSOD Dataset [35]:

This dataset includes 976 high-resolution remote sensing images, each with a resolution of 800 × 800 pixels. It is divided into four categories: Aircraft, Oil tanks, Overpasses, and Playgrounds, totaling 7400 object instances.

### 4.2. Implementation Details

Our proposed EDM-Net is trained for 300 epochs using a single NVIDIA GeForce RTX 4090D (24 GB). The training environment is detailed in Table 1. The initial learning rate, momentum, weight decay, and batch size are set to 0.0001, 0.9, 0.0001, and 8, respectively.

During training, according to the official original paper settings for the DIOR dataset, the ratio of the training, validation, and test sets is 0.25:0.25:0.5. The dataset is partitioned into 5865 training images, 5866 validation images, and 11,732 testing images. For the NWPU VHR-10 dataset, we focus on 650 positive samples containing object annotations. Using a fixed random seed of 0, we split these images into training, validation, and test sets at a ratio of 7:1:2. The 150 negative images were not used for AP evaluation because they do not contain annotated object instances. For the RSOD dataset, using a fixed random seed of 0, the entire dataset is partitioned into training, validation, and test sets at a ratio of 7:1:2 to ensure a balanced distribution for model training and evaluation.

### 4.3. Evaluation Metrics

To objectively quantify model performance, this paper adopts Average Precision (AP) and mean Average Precision (mAP) as the core evaluation metrics. Specifically, mAP50 is defined as the mean AP across all categories calculated at an Intersection over Union (IoU) threshold of 0.5; mAP50:95 is defined as the average AP calculated across multiple IoU thresholds from 0.5 to 0.95 (in steps of 0.05). All experimental results follow the official evaluation protocols of each dataset to ensure fair comparisons with existing methods. Precision (P) and Recall (R) are calculated as follows:

(12) $$Precision=\frac{TP}{TP+FP}$$

(13) $$Recall=\frac{TP}{TP+FN}$$

where TP, FP, and FN represent True Positives, False Positives, and False Negatives, respectively. AP is calculated from the area under the Precision–Recall curve:

(14) $$AP=\int_{0}^{1}P\left(R\right)dR$$

(15) $$mAP=\frac{1}{N}\sum_{i=1}^{N}\;AP_{i}$$

Furthermore, to further evaluate the model’s detection performance on objects of different scales, we introduce subdivided evaluation metrics based on target pixel area size: AP_S_ evaluates the average precision for small objects (pixel area < 32 × 32); AP_M_ evaluates the average precision for medium objects (32 × 32 ≤ pixel area ≤ 96 × 96); AP_L_ evaluates the average precision for large objects (pixel area > 96 × 96).

## 5. Results and Analysis

### 5.1. Comparison with State-of-the-Art Methods

We comprehensively compared EDM-Net with detectors that have exhibited outstanding performance in remote sensing and natural image domains in recent years. This covers CNN single-stage (YOLO series), two-stage (Faster R-CNN), Anchor-Free (FCOS), and Transformer-based end-to-end models (DETR, DINO, RT-DETR).

For fair comparison, all baseline models re-trained in this study followed the same data split, input size, training epochs, augmentation policy, and evaluation script as EDM-Net. Results imported from published papers are marked with their corresponding references. Because some published results may use different splits, input resolutions, or training details, we treat them as reference-level comparisons. The causal claims about the effectiveness of EDM-Net are mainly supported by the re-trained baselines, ablation studies, and scale-specific analyses conducted under the same protocol. Results marked with ‘*’ in the tables are re-trained under identical experimental settings. Results without this mark are directly cited from the corresponding references and are provided for reference only.

**Figure 7:**
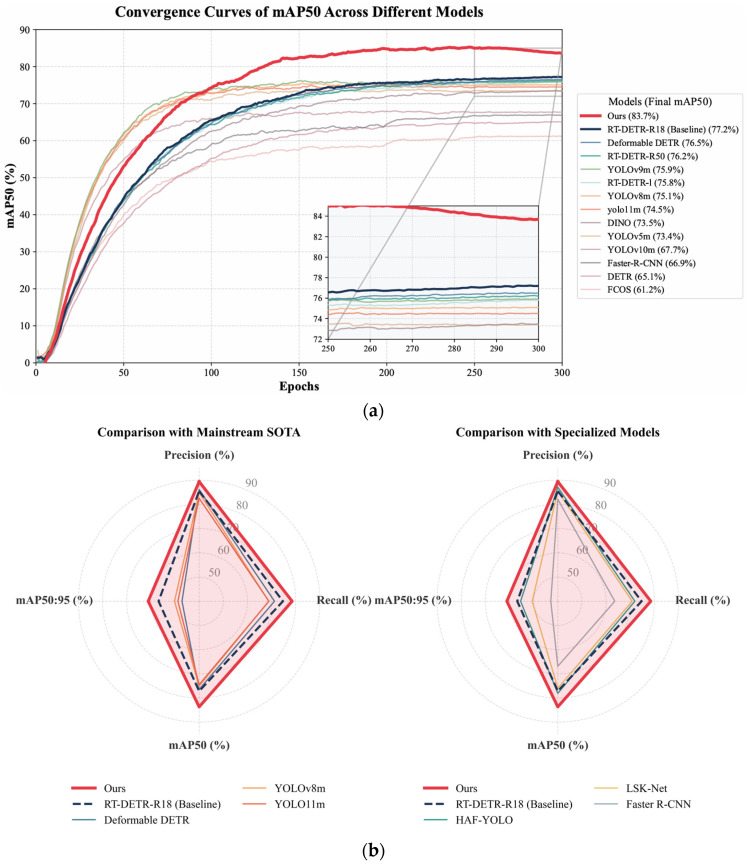
Comparative analysis of convergence curves and comprehensive performance between our method and other models. (**a**) Comparison of training convergence curves. (**b**) (**Left**) Performance comparison with mainstream SOTA models; (**Right**) performance comparison with task-specific specialized models.

**Figure 8:**
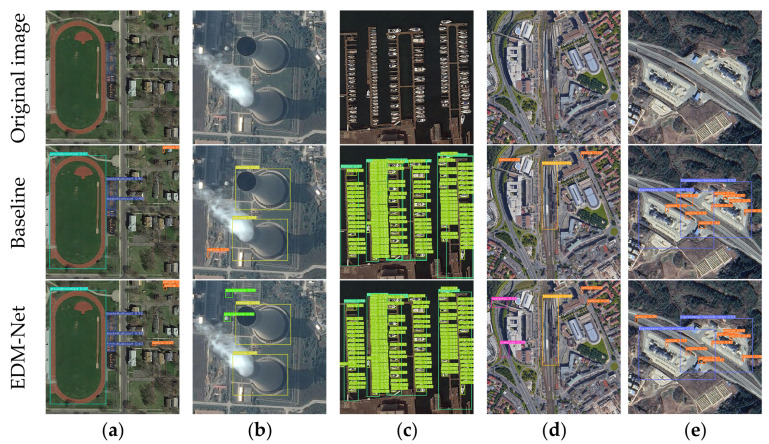
Qualitative comparison results of EDM-Net on the DIOR dataset. (**a**) Large objects, (**b**) medium objects, (**c**) small objects, (**d**) objects in complex backgrounds, (**e**) multi-scale objects within the same image.

(1) Experiment Results on DIOR:

As shown in Table 2, EDM-Net achieved an overall mAP50 of 83.7% on the DIOR test set. It not only outperformed the baseline model with an absolute advantage of 6.5% but also obtained optimal performance in 11 out of the 20 total categories. Notably, the performance improvement of EDM-Net is particularly significant when facing challenging categories characterized by typical multi-scale and dense distributions. For instance, in the Harbor (76.8%) and Overpass (76.6%) categories, it leads the second-best method by up to 7.2 percentage points. It also achieved significant gains of 3.7% and 3.1% in the Ground track field and Ship categories, respectively. These significant results demonstrate the superiority of the proposed model in handling the synergistic detection of large, medium, and small-scale objects. Combined with the qualitative comparison results in Figure 7 and Figure 8, this further intuitively showcases EDM-Net’s precise and robust detection capability in complex remote sensing scenes.

Figure 7a shows the mAP50 convergence curves of different models trained with the same configuration on the DIOR dataset. EDM-Net not only converged significantly faster than the comparative methods in the early training stage (first 50 epochs) but also exhibited a clear accuracy advantage in the late training stage (250–300 epochs). This indicates that the dynamic sparse activation in ES-MoE offers a better start for optimization. It helps the model avoid gradient shocks caused by background noise. Figure 7b quantifies a comprehensive comparison of EDM-Net against other models across four key evaluation metrics: Precision, Recall, mAP50, and mAP50:95. When compared with mainstream SOTA and specialized models in the remote sensing domain, the results show that EDM-Net exhibits outstanding and balanced comprehensive performance across all dimensions.

The visual comparison results in Figure 8 further highlight the superior performance of EDM-Net in object detection. Compared to the baseline model, the baseline method exhibits obvious missed detections or feature confusion when processing regions with densely distributed small objects (Figure 8c) and complex backgrounds (Figure 8d). In contrast, EDM-Net demonstrates a strong ability to capture fine-grained object clues even under the challenges of severe background interference or the coexistence of multi-scale objects (Figure 8e). This leap in visual performance originates from EDM-Net’s unique multi-expert dynamic perception and hierarchical feature enhancement mechanisms, which synergistically filter background noise and generate more discriminative feature representations for tiny object perception. As demonstrated by the visualizations, EDM-Net consistently identifies the vast majority of objects in cluttered remote sensing environments. Conversely, the baseline method, limited by the lack of spatial inductive bias, tends to ignore low-contrast or highly overlapping objects.

**Figure 9:**
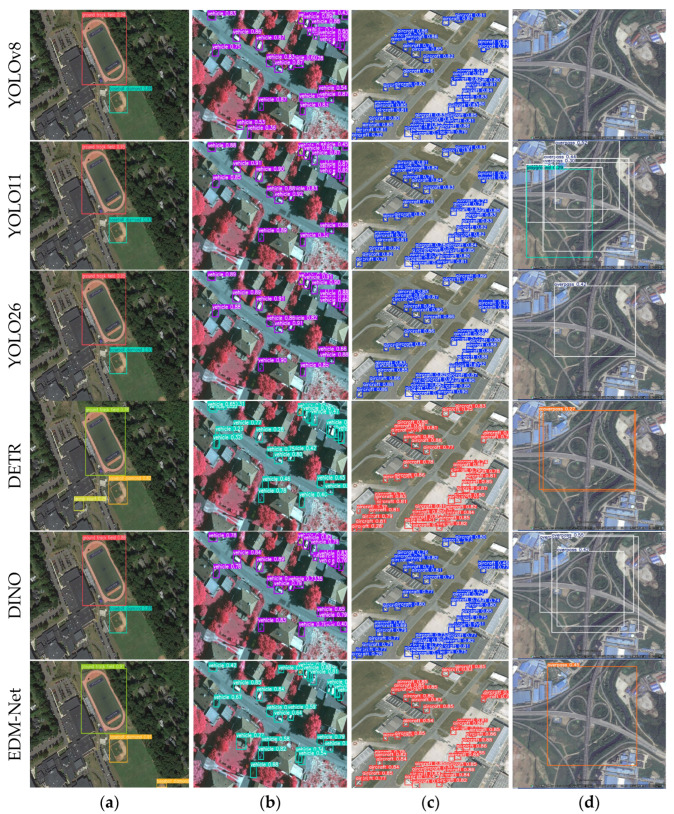
Some representative predictions of EDM-Net on the NWPU VHR-10 and RSOD datasets. (**a**,**b**) Results on NWPU VHR-10. (**c**,**d**) Results on RSOD. These represent large objects, small objects in dense backgrounds, and objects in complex backgrounds, respectively.

(2) Experiment Results on NWPU VHR-10:

To further evaluate EDM-Net, we conducted tests on the NWPU VHR-10 dataset. The experiment results are illustrated in Table 3. Our proposed EDM-Net achieved the best mAP50 performance of 95.6%, outperforming the baseline model (92.1%) by 3.5%. Specifically, EDM-Net achieved optimal accuracy in 2 out of the 10 categories. Compared with the second-best models, the mAP improved by 0.3% and 1.1%, respectively. Notably, in the C1 (Airplane) category, EDM-Net achieved an AP of 100%, and the APs for all other categories were above 85%, demonstrating the superiority of EDM-Net. Figure 9a,b show partial visual detection results on the NWPU VHR-10 dataset.

(3) Experiment Results on RSOD:

Table 4 demonstrates the comparison results on the RSOD dataset. EDM-Net achieved an mAP50 of 95.8% on this dataset, representing a 3.2% improvement over the baseline (92.6%). Notably, the model reached robust performance across all categories. Particularly noteworthy is that the AP for the Oil tanks category is 0.4% higher than the second-place result, demonstrating EDM-Net’s effectiveness in dense small object detection. Figure 9c,d presents the visual detection results.

We conducted an in-depth cross-dataset visual analysis using representative samples from the NWPU VHR-10 and RSOD datasets. As shown by the multi-scale coexistence results in the first column of Figure 9, EDM-Net can reliably perform synergistic detection when confronted with extreme scale variations. Furthermore, even in restricted scenarios characterized by significantly degraded visual cues and extreme perspective distortions, EDM-Net continues to effectively identify the highly challenging instances shown at the bottom of the first column in Figure 9, whereas comparative models like DETR and YOLO11 exhibit obvious missed detections. The third column highlights the advantages of our method in dense small-object scenarios: competing methods such as YOLO26 and DINO miss numerous overlapping instances due to feature adhesion, while EDM-Net demonstrates strong robustness with only isolated omissions. Additionally, the second and fourth columns show that under complex background interference and extremely low visibility, EDM-Net still outputs clear and complete detection boundaries. Conversely, baseline models such as YOLOv8 and DINO produce severe fragmented false positives or completely fail to capture the targets in these areas. These intuitive observations collectively verify that the core advantages of EDM-Net are particularly prominent in extreme situations involving high density, drastic scale variations, and complex backgrounds, proving that it can effectively scale under various demanding remote sensing imaging conditions and maintain strong generalization capabilities across different datasets.

(4) Cross-Scale Performance Analysis:

To quantify the model’s multi-scale processing capability, Table 5 compares the accuracy of EDM-Net and the baseline model across objects of varying sizes. The data indicates that EDM-Net achieved steady growth across the AP_S_, AP_M_, and AP_L_ metrics on all three datasets. Especially on the DIOR dataset, where small objects account for a high 51.7%, EDM-Net increased AP_S_ from the baseline’s 22.4% to 26.8% (a 19.6% relative improvement). This confirms that the adaptive-scale context learning strategy proposed in this paper does not come at the expense of large object semantics, but rather achieves an effective complementation of multi-granularity features.

**Figure 10:**
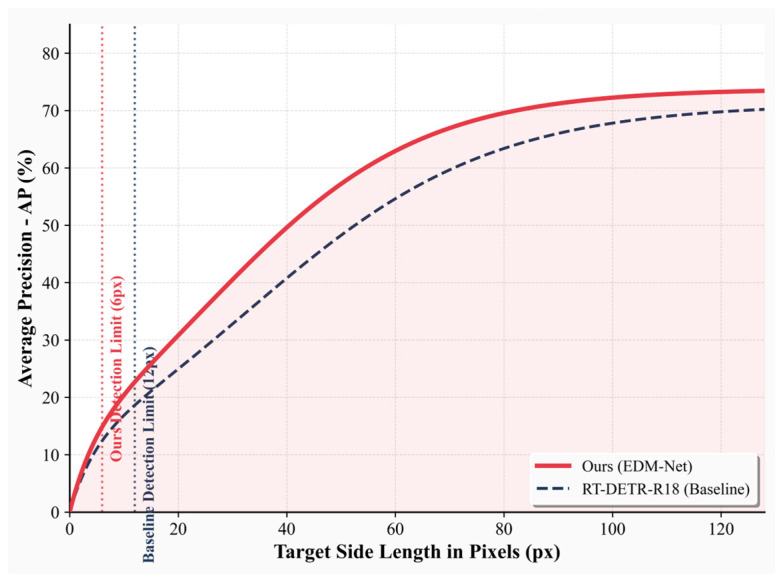
The red solid curve corresponds to EDM-Net, and the dark blue dashed curve corresponds to the baseline RT-DETR-R18. The two vertical dashed lines mark the minimum detectable size boundaries of the two models (defined as the target side length when AP50 reaches 20%): 6 pixels for EDM-Net and 12 pixels for the baseline.

To further characterize the continuous relationship between target pixel size and detection performance, we divide all annotated instances into continuous intervals by target side length and calculate the corresponding AP50 score for each interval. Figure 10 presents the variation trend of average precision with target side length for both EDM-Net and the baseline model. We define the minimum detectable size as the side length corresponding to an AP50 of 20%, which marks the size boundary where the model can stably capture valid target semantic features.

As shown in Figure 10, the AP of both models increases monotonically as the target size grows, and EDM-Net consistently outperforms the baseline across all size intervals. In terms of the detection boundary, EDM-Net achieves a minimum detectable side length of 6 pixels, while the baseline RT-DETR-R18 has a minimum detectable side length of 12 pixels. This indicates that EDM-Net can capture effective target features at half the size of the baseline, exhibiting a prominent advantage in extremely small target detection.

### 5.2. Ablation Study

We conducted ablation studies to examine whether the performance gains of EDM-Net come from the proposed extraction–interaction–fusion design. ES-MoE, MSAF, and DMIFI were removed individually and combined cumulatively to evaluate their separate and joint contributions. This remove/add ablation setting directly tests whether each module contributes to the final model under the same training and evaluation protocol.

**Figure 11:**
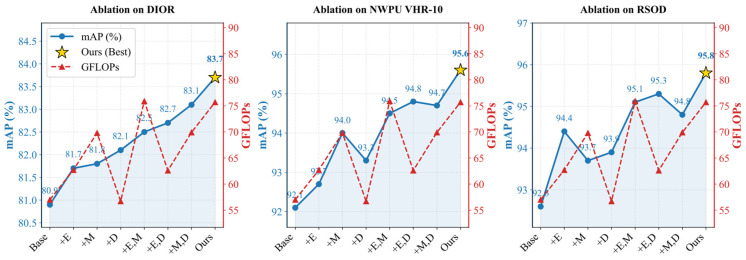
Ablation experiment results. The blue solid line represents mAP50 accuracy, the red dashed line represents GFLOPs computational load, and the star represents the complete EDM-Net model. E, M, and D represent the ES-MoE, MSAF, and DMIFI modules, respectively. The stacking of each module continuously improves detection accuracy, and the complete model achieves optimal performance across all three datasets.

**Figure 12:**
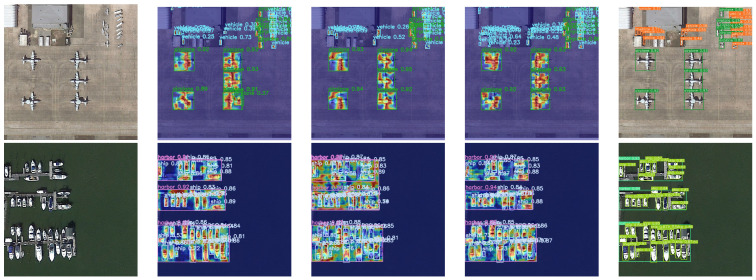

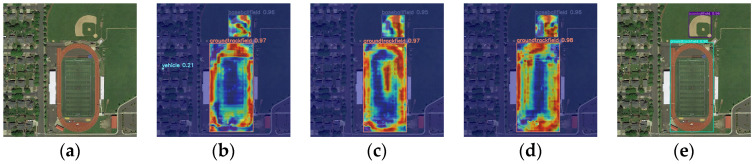
Comparative visualization of model heat map effects: (**a**) Original image, (**b**) ES-MoE extraction, (**c**) MSAF, (**d**) heat map visualization, (**e**) detection results. These represent dense small objects and high similarity between categories, respectively. The heat map uses pseudo-color to represent feature response intensity, with blue indicating low response and red indicating high response.

The ablation experiment results are summarized in Table 6, Table 7 and Table 8. √ indicates the module is used, and × indicates the module is not used. Figure 11 displays the model’s computational complexity and detection accuracy. Furthermore, Figure 12 presents the feature map visualization results. To further quantify the impact of each module on practical inference efficiency. We report three practical inference metrics: average single-image latency, frames per second (FPS), and peak GPU memory usage, as summarized in Table 9. A more in-depth analysis of the overall performance–complexity trade-off is provided in Section 6.4.

As shown in Figure 11, the detection performance of the baseline model steadily improved when the proposed modules were introduced, either individually or in pairs. The final integration of all three modules collectively enhanced feature extraction, scale adaptation, and context fusion, enabling the complete EDM-Net to achieve the highest detection accuracies of 83.7%, 95.6%, and 95.8% on the three datasets, respectively. This demonstrates the strong independence and synergy among the modules. Regarding computational complexity, although the ES-MoE module brought additional overhead, the module combinations involving ES-MoE often exhibited higher efficiency. Overall, the complete EDM-Net achieved a remarkable leap in accuracy with a moderate increase in computational load compared to the baseline.

### 5.3. Visualization Results of Feature Extraction and Fusion

To further demonstrate the specific roles of each module during the feature extraction and fusion process, we visualized and compared feature heat maps at different stages in Figure 12. In Figure 12b, after extraction by the ES-MoE module, the network initially captured salient features of the targets; however, the feature map still contained some background interference, such as ground textures of airport aprons or water surfaces in harbors. Moreover, in scenes with densely arranged ships, there was a certain degree of feature adhesion among adjacent targets, challenging subsequent precise localization. In Figure 12c, after introducing the MSAF module for multi-scale adaptive fusion, background noise was significantly suppressed. It can be seen that the features in target regions for airplanes, athletic fields, and dense ships were effectively enhanced, and the responses became much more focused. The final heat map visualization in Figure 12d indicates that the output features became clearer and more precise. Especially in areas densely packed with harbor ships, the network could substantially reduce feature confusion between adjacent ships, clearly separating the vast majority of individual entities. Combined with the final detection results in Figure 12e, these visualization results strongly prove that the proposed modules not only effectively reduce interference from complex backgrounds and environmental noise but also significantly boost the feature expression of multi-scale and dense objects in remote sensing images, thereby effectively improving the model’s final object detection performance.

## 6. Discussion

### 6.1. Interpretation of Multi-Dataset Superiority and Ablation Insights

EDM-Net achieves strong performance across DIOR, NWPU VHR-10, and RSOD under the experimental protocols used in this study. The gains are not explained solely by adding more parameters. Instead, the ablation results suggest that the three modules improve different parts of the detection pipeline. ES-MoE mainly improves the extraction of scale-sensitive features, DMIFI strengthens local spatial interaction after global attention, and MSAF reduces semantic mismatch during multi-scale aggregation. Their cumulative effect is most visible in scenes containing dense small objects and complex backgrounds.

Our synergistic extraction–interaction–fusion pipeline systematically dismantles these challenges. As evidenced by the ablation studies, the isolated addition of the ES-MoE module yields the most substantial accuracy leap for small objects. Mechanistically, ES-MoE replaces inefficient uniform computation with a dynamic routing paradigm. By allocating experts based on spatial complexity, it suppresses the noise of vast backgrounds while preserving high-frequency details for tiny targets.

Furthermore, to address the dense clusters prominent in RSOD, the DMIFI module re-injects 2D spatial inductive bias to overcome the boundary over-smoothing defect of standard Transformers. By adaptively reconstructing local textures through dynamic convolutions, it enables the network to accurately separate adjacent instances. Finally, the MSAF module effectively neutralizes the semantic dilution typically observed in standard FPNs. By decoupling macroscopic context from local structures via parallel attention branches, MSAF ensures that the deep semantic abstractions of large objects do not overwrite the shallow geometric details of tiny targets, explaining our model’s robust performance across massive scale spans.

### 6.2. Category-Specific Bottlenecks and Failure Mechanisms

Despite leading overall metrics, an objective error analysis reveals performance bottlenecks in specific categories, most notably for targets like Bridge or Overpasses. This limitation primarily arises from the difficulty in modeling continuous topology for objects with high aspect ratios.

Linear structures often stretch across complex backgrounds. Gradient variations in these adjacent backgrounds cause feature aliasing. During the interaction and fusion stages, this aliasing interferes with the extraction process, occasionally fragmenting continuous linear features into disconnected segments and causing missed detections. Furthermore, under scenarios with low resolution and low contrast, the blurred input information causes the dynamic routing of the ES-MoE module to produce activation oscillations, making it difficult for the network to stably extract details. This indicates a need for geometrically resilient representations when handling structural distortions.

### 6.3. Research Contributions and Future Perspectives

The contribution of this research lies in establishing a unified feature processing paradigm that integrates scale-adaptive extraction, boundary sharpening, and semantic alignment. EDM-Net provides a structural reference for the remote sensing community. Practically, its precision against complex backgrounds makes it applicable for real-world tasks, including urban infrastructure monitoring, maritime traffic surveillance, and disaster damage assessment.

However, edge deployment reveals current limitations. While structural re-parameterization optimizes inference speed, the parameter scale of the network still presents optimization space for low-power edge platforms. In the future, work will focus on: (1) investigating lightweight strategies such as model pruning and quantization to meet edge-computing constraints; and (2) incorporating self-supervised pre-training using unlabeled remote sensing data to enhance the model’s robustness against degraded imaging conditions.

### 6.4. Efficiency Analysis and Performance–Complexity Trade-Offs

As shown in Table 9, the full EDM-Net achieves an inference speed of 40.6 FPS, which still meets the real-time requirement for most remote sensing applications despite the increase in parameters and computation. From the perspective of individual modules, the efficiency characteristics of the three modules are consistent with their design goals:

The ES-MoE module introduces the largest parameter increment (nearly doubling the baseline parameters), but its actual inference speed drop (19.8%) is much lower than the parameter growth rate. This verifies that the sparse expert activation mechanism effectively controls actual computation overhead—only a small number of experts are activated per input during inference, so the computational load does not increase linearly with the number of parameters.

The DMIFI module has a negligible impact on inference speed (only 2.1% FPS reduction) with almost no additional parameters. This is because the dynamic spatial mixing operation based on depthwise convolution is highly optimized in modern deep learning frameworks, and the computational overhead is far lower than global self-attention.

The MSAF module benefits from structural re-parameterization: its multi-branch topology is completely merged into a single convolution operator during inference, so it does not introduce extra inference branches. Its main overhead comes from the channel projection and attention branches, which is within an acceptable range.

Overall performance–complexity trade-off: We discuss the rationality of the accuracy–speed trade-off of the full model, and clarify applicable scenarios that prioritize accuracy and scenarios that have stricter efficiency requirements.

### 6.5. Detection Boundary Analysis for Extremely Small Targets

The detection boundary comparison in Figure 10 demonstrates that EDM-Net effectively reduces the minimum detectable size of remote sensing targets from 12 pixels to 6 pixels. This improvement in the extreme small-size regime is not achieved by simply increasing parameters, but stems from the synergistic effect of the three proposed modules across the entire feature processing pipeline.

First, the ES-MoE module embedded in the backbone adaptively activates small-kernel expert branches for tiny target regions, avoiding feature smoothing caused by large receptive fields on extremely small objects, and preserves fine-grained texture details at the feature extraction stage. Second, the DMIFI module supplements 2D local spatial inductive bias after global self-attention, and reconstructs boundary features of tiny targets through dynamic spatial mixing, which alleviates the boundary over-smoothing problem of the standard Transformer encoder. Third, the MSAF module performs semantic alignment before cross-scale feature fusion, reducing the dilution of tiny target features during the aggregation of deep semantic and shallow detail features, ensuring that features of 6-pixel-level targets can be retained to the final prediction layer.

From an application perspective, the forward shift of the detection boundary enables the model to identify smaller ground targets under the same imaging resolution, which has practical value for scenarios such as ultra-high-altitude remote sensing reconnaissance and small target search and rescue.

## 7. Conclusions

In this article, we introduce EDM-Net, a synergistic multi-scale object detection network tailored for remote sensing images, which is composed of ES-MoE, DMIFI, and MSAF. These components play pivotal roles in the highly coupled extraction–interaction–fusion pipeline. The effectiveness of detecting dense tiny objects amidst extreme scale variations and complex background interference holds particular significance in our study. Additionally, comprehensive experiments and ablation studies underscore the complementary advantages and synergistic effects offered by these three key components. However, the proposed method is not without disadvantages. Although EDM-Net achieves clear accuracy improvements over the re-trained baseline models under the same experimental protocol, the overall network is still not sufficiently lightweight, leaving room for further optimization when targeting ultra-low-power edge devices.

In the future, our endeavors will focus on researching lightweight strategies, such as structured model pruning and quantization, to maintain high detection accuracy while further reducing computational complexity. Meanwhile, these efforts will aim to meet the strict demands of real-time deployment on various resource-constrained mobile platforms, such as airborne unmanned aerial vehicles (UAVs) and surveillance systems.

## Figures and Tables

**Table 1:** Experimental training environment.

Component	Configuration
Operating System	Ubuntu 22.04 LTS
CPU	Intel Core i9-12900K @ 3.20 GHz
Deep Learning Framework	PyTorch 2.2.1
Programming Language	Python 3.10.12
GPU	NVIDIA GeForce RTX 4090D (24 GB)
CUDA Version	CUDA 12.2

**Table 2:** Quantitative comparison results on the DIOR dataset. The best and second-best results for each category are highlighted in bold and blue, respectively. Models marked with * are re-trained under identical experimental settings for fair comparison.

Method	C1/C11	C2/C12	C3/C13	C4/C14	C5/C15	C6/C16	C7/C17	C8/C18	C9/C19	C10/C20	mAP50
YOLOv5m *	92.5/84.2	69.8/62.9	91.9/57.9	87.2/90.4	40.8/89.1	78.6/82.7	55.1/92.3	83.2/48.5	65.9/46.8	69.2/78.2	73.4
YOLOv8m *	93.4/84.4	74.2/64.2	92.5/60.6	86.9/90.5	42.2/91.6	78.0/82.5	58.5/92.3	86.5/55.6	65.4/48.1	73.9/79.6	75.1
YOLOv9m * [23]	93.9/85.8	73.2/63.2	92.8/62.0	87.6/90.6	44.8/91.6	80.1/83.3	58.5/92.8	86.2/56.2	66.5/49.1	78.8/82.0	75.9
YOLOv10m * [24]	93.2/79.3	49.1/52.9	91.2/47.7	85.7/91.1	35.2/82.0	72.8/84.5	39.4/92.3	70.5/31.8	63.0/50.4	61.5/80.5	67.7
YOLO11m * [25]	93.8/86.4	67.1/61.6	92.2/59.7	87.1/91.0	42.1/90.9	78.4/84.1	56.1/92.9	83.5/52.4	64.7/49.4	75.4/80.3	74.5
YOLO26m * [27]	93.5/84.6	74.5/64.5	92.4/61.1	87.2/90.6	42.5/92.3	77.1/82.8	58.7/92.0	86.4/55.8	65.8/48.5	74.1/80.1	75.0
Faster-R-CNN [19]	81.1/78.3	76.9/43.3	89.8/58.4	80.5/68.5	45.1/89.1	81.7/59.0	63.4/81.1	80.9/60.2	63.2/**77.8**	73.3/79.3	66.9
FCOS [58]	73.5/68.7	68.0/46.3	69.9/51.1	85.1/72.2	34.7/59.8	73.6/64.6	49.3/81.2	52.1/42.7	47.6/42.2	67.2/74.8	61.2
DETR * [30]	88.5/75.1	45.2/45.2	88.2/40.2	78.1/85.1	36.1/78.5	70.2/78.1	52.2/88.4	65.1/45.4	65.4/38.2	67.1/72.4	65.1
Deformable DETR [31]	92.4/68.5	64.6/69.6	87.6/69.4	82.3/74.6	42.5/88.5	72.6/73.5	63.3/77.4	79.1/68.2	54.3/71.3	77.3/72.3	76.5
DINO * [32]	93.1/82.3	70.2/63.5	91.8/58.1	85.1/90.1	40.1/89.1	78.5/83.1	55.8/92.5	83.8/49.1	65.3/49.6	70.1/78.1	73.5
HAF-YOLO [59]	82.7/81.7	**89.8**/68.4	85.8/64.2	90.2/91.4	48.7/80.2	79.2/75.1	72.4/92.3	88.8/67.0	69.6/51.3	**86.9**/86.5	78.1
CFBA-FPN [60]	90.1/87.8	81.5/63.1	94.3/64.9	**91.8**/82.7	**55.2**/92.2	92.1/83.5	65.2/**95.9**	85.6/57.7	**86.8**/61.8	83.2/56.3	78.6
DINO-SE-SD [61]	71.9/83.5	89.0/53.4	78.7/64.6	87.7/76.4	51.9/76.9	81.5/73.6	68.9/87.9	89.2/67.5	78.9/54.6	79.9/**90.4**	75.3
TBNet [62]	64.9/83.6	86.8/56.4	76.6/64.9	89.2/79.1	50.6/77.8	80.0/58.1	**74.3**/88.2	86.4/**69.4**	74.7/44.6	82.5/87.0	73.6
LSK-Net [63]	91.0/74.1	77.8/60.0	92.9/63.9	83.8/78.5	53.4/90.3	**92.2**/81.8	65.1/90.7	68.7/61.3	60.0/73.6	83.8/76.3	76.0
GLNet [6]	62.9/83.0	83.2/51.8	72.0/62.6	81.1/72.0	50.5/75.3	79.3/53.7	67.4/81.3	86.2/65.5	70.9/43.4	81.8/89.2	70.7
CoF-Net [8]	84.0/83.2	85.3/57.4	82.6/62.2	90.0/82.9	47.1/77.6	80.7/68.2	73.3/89.9	89.3/68.7	74.0/49.3	84.5/85.2	75.8
SFSANet [43]	87.8/78.6	83.3/62.9	87.8/63.6	86.7/90.2	48.8/83.4	78.5/79.4	68.2/83.8	85.0/61.5	76.5/67.9	73.2/84.7	76.6
S^2^EKD [64]	66.9/76.8	79.1/44.9	75.3/56.8	87.5/72.6	41.3/71.9	76.2/61.2	64.1/84.7	75.4/63.0	58.5/42.9	78.8/83.1	68.1
ACI-former [65]	91.8/69.4	68.2/67.5	84.6/68.4	83.6/76.3	40.7/86.2	73.6/74.6	68.2/81.4	76.4/64.6	58.3/69.4	78.2/74.8	76.7
RT-DETR-L * [51]	92.8/84.5	69.5/63.2	91.5/58.5	87.5/90.5	41.2/89.5	79.1/82.5	60.5/92.1	88.6/49.2	71.2/47.1	75.5/78.5	75.8
RT-DETR-R50 * [51]	92.2/90.5	75.4/61.4	90.6/60.4	87.5/90.1	50.3/94.8	80.1/**88.2**	67.1/91.2	**90.1**/54.8	71.5/50.6	78.8/83.1	76.2
RT-DETR-R18 * [51]	88.5/85.9	78.7/56.9	88.9/61.5	86.1/87.2	51.0/86.1	74.2/83.1	66.1/86.5	82.9/53.4	78.2/70.2	81.7/88.5	77.2
Ours (EDM-Net)	**95.6**/**94.2**	88.2/**76.8**	**95.6**/**76.6**	89.1/**94.5**	**55.2**/**95.3**	88.2/85.5	71.1/92.1	89.1/63.5	85.5/64.4	83.6/89.7	**83.7**

**Table 3:** Quantitative comparison results on the NWPU VHR-10 dataset. The best and second-best results for each category are highlighted in bold and blue, respectively. Models marked with * are re-trained under identical experimental settings for fair comparison.

Method	C1	C2	C3	C4	C5	C6	C7	C8	C9	C10	mAP
YOLOv5m *	99.5	97.4	62.8	96.1	97.4	91.4	97.9	86.7	90.7	90.1	91.0
YOLOv8m *	99.5	96.4	45.4	97.7	99.3	94.5	97.1	91.6	96.8	88.7	90.7
YOLOv9m * [23]	96.3	96.2	67.5	95.7	89.9	92.1	93.6	88.2	80.6	88.5	89.1
YOLOv10m * [24]	94.1	93.6	68.1	92.0	90.2	88.9	95.7	86.5	83.2	88.3	88.1
YOLO11m * [25]	99.5	**97.8**	61.2	98.3	99.4	92.5	95.9	88.6	97.2	89.4	91.9
YOLO26m * [27]	99.5	91.3	64.5	96.5	**99.5**	97.7	96.5	91.0	95.6	86.0	91.1
DETR * [30]	96.3	85.0	69.0	97.1	96.5	87.5	99.3	93.6	86.0	85.3	88.7
DINO * [32]	97.2	94.1	96.8	96.1	93.0	95.2	97.2	91.1	72.1	89.5	92.1
FCOS [58]	98.9	90.6	93.7	95.6	93.6	92.6	99.9	79.1	82.3	90.7	91.7
Deformable DETR * [31]	97.5	88.9	91.1	93.3	89.4	87.6	94.4	83.7	73.7	79.1	87.9
CoF-Net [8]	**100**	90.9	96.1	98.8	91.1	95.8	**100**	91.4	89.7	90.8	94.5
GLNet [6]	**100**	84.4	81.6	98.5	88.2	**100**	97.2	88.4	90.9	88.7	91.8
SCRDet [11]	**100**	89.4	97.2	97.0	83.2	87.5	99.2	**99.4**	74.5	90.1	91.8
DINO-SE-SD [61]	99.7	93.0	96.4	95.1	96.6	96.5	**100**	93.6	69.9	87.2	92.8
SFSANet [43]	**100**	87.7	74.4	**99.4**	95.9	93.9	98.9	97.1	80.9	95.0	92.3
SAFF-DETR [54]	99.3	81.9	92.2	98.9	96.0	94.3	99.4	87.3	**98.6**	88.1	93.3
HAF-YOLO [59]	98.8	76.8	87.1	97.1	92.2	60.6	97.1	92.5	63.3	84.4	85.0
TBNet [62]	**100**	95.09	**97.35**	97.35	97.55	98.96	**100**	94.64	80.77	92.71	95.44
RT-DETR-L * [51]	97.8	97.2	68.7	93.3	97.1	86.0	95.5	82.7	76.8	82.7	87.8
RT-DETR-R50 * [51]	99.5	95.6	70.4	94.1	98.2	89.6	99.5	91.9	**98.6**	90.4	92.1
RT-DETR-R18 * [51]	96.3	93.7	79.2	92.1	96.5	93.2	97.3	92.1	88.5	90.8	92.1
Ours (EDM-Net)	**100**	95.5	85.1	96.4	99.0	99.5	97.0	91.2	95.2	**96.1**	**95.6**

**Table 4:** Quantitative comparison results on the RSOD dataset. The best and second-best results for each category are highlighted in bold and blue, respectively. Models marked with * are re-trained under identical experimental settings for fair comparison.

Method	Aircraft	Oil Tanks	Overpasses	Playgrounds	mAP
YOLOv5m *	92.9	99.3	76.8	99.5	92.1
YOLOv8m *	92.5	99.4	74.7	99.5	91.5
YOLOv9m * [23]	87.9	99.4	55.1	93.5	84
YOLOv10m * [24]	93.1	99.4	68.5	99	90
YOLO11m * [25]	90.8	99.2	65.4	93.2	87.1
YOLO26m * [27]	92.3	99.4	77.7	98.6	92.0
DETR * [30]	90.9	98.5	76.7	93.5	90.2
DINO * [32]	93.0	95.9	80.2	96.1	91.2
Faster RCNN [19]	70.84	90.19	78.74	98.09	84.5
EfficientDet [48]	85.7	92.6	54.6	98.2	82.8
Sig-NMS [66]	80.6	90.6	87.4	99.1	89.4
CANet [67]	91.7	97.0	94.1	97.9	95.2
CGA-YOLO [68]	95.4	96.6	69.7	99.5	90.3
AGMF-Net [69]	96.02	99.02	82.43	99.7	94.3
YOLO-SBA [70]	**97.56**	96.22	85.81	99.31	94.7
TBNet [62]	95.24	96.76	**95.92**	**100**	**96.98**
RT-DETR-L * [51]	90.8	99.0	54.3	99.5	85.9
RT-DETR-R50 * [51]	89.9	99.4	42.7	98.6	82.6
RT-DETR-R18 * [51]	92.1	98.6	86.9	94.1	92.6
Ours (EDM-Net)	94.2	**99.8**	89.2	99.6	95.8

**Table 5:** Comparison of AP_S_, AP_M_, and AP_L_ metrics between EDM-Net and the baseline model across the three datasets. ↑ indicates improvement compared with the baseline model.

Dataset	Size Distribution	AP_S_		AP_M_		AP_L_	
		Baseline	Ours	Baseline	Ours	Baseline	Ours
DIOR	51.7/30.2/18.1	22.4	26.8 (↑ 4.4)	52.6	56.2 (↑ 3.6)	68.7	71.1 (↑ 2.4)
NWPU VHR-10	4.3/77.6/18.1	30.9	36.4 (↑ 5.5)	53.1	58.6 (↑ 5.5)	63.5	65.7 (↑ 2.2)
RSOD	22.1/62.8/15.1	33.5	37.8 (↑ 4.3)	65.6	68.3 (↑ 2.7)	72.1	73.2 (↑ 1.1)

**Table 6:** Ablation experiment results on the DIOR dataset. √ indicates the module is adopted; × indicates the module is not adopted; ↑ indicates improvement compared with the baseline.

Baseline	ES-MoE	MSAF	DMIFI	P (%)	R (%)	mAP50 (%)	mAP50:95 (%)	Params (M)	GFLOPs
√	×	×	×	85.5	74.7	77.2	56.7	19.88	57.0
√	√	×	×	88.9	76.0	81.7 (↑ 4.5)	58.6	39.81	62.7
√	×	√	×	88.0	77.4	81.8 (↑ 4.6)	59.1	23.71	69.8
√	×	×	√	87.7	77.6	82.1 (↑ 4.9)	58.9	19.94	56.7
√	√	√	×	89.2	77.9	82.5 (↑ 5.3)	59.5	43.74	75.9
√	√	×	√	88.9	78.4	82.7 (↑ 5.5)	59.8	39.90	62.6
√	×	√	√	88.5	79.0	83.1 (↑ 5.9)	60.5	23.97	69.9
√	√	√	√	89.4	78.5	83.7 (↑ 6.5)	61.1	43.81	75.7

**Table 7:** Ablation experiment results on the NWPU VHR-10 dataset. √ indicates the module is adopted; × indicates the module is not adopted; ↑ indicates improvement compared with the baseline.

Baseline	ES-MoE	MSAF	DMIFI	P (%)	R (%)	mAP50 (%)	mAP50:95 (%)	Params (M)	GFLOPs
√	×	×	×	86.9	82.4	92.1	59.7	19.88	57.0
√	√	×	×	92.0	84.6	92.7 (↑ 0.6)	67.4	39.81	62.7
√	×	√	×	91.9	86.3	94.0 (↑ 1.9)	63.8	23.71	69.8
√	×	×	√	91.5	83.8	93.3 (↑ 1.2)	63.5	19.94	56.7
√	√	√	×	92.5	86.1	94.5 (↑2.4)	67.8	43.74	75.9
√	√	×	√	93.1	86.8	94.8 (↑ 2.7)	67.6	39.90	62.6
√	×	√	√	92.8	86.4	94.7 (↑ 2.6)	66.2	23.97	69.9
√	√	√	√	94.1	97.2	95.6 (↑ 3.5)	68.5	43.81	75.7

**Table 8:** Ablation experiment results on the RSOD dataset. √ indicates the module is adopted; × indicates the module is not adopted; ↑ indicates improvement compared with the baseline.

Baseline	ES-MoE	MSAF	DMIFI	P (%)	R (%)	mAP50 (%)	mAP50:95 (%)	Params (M)	GFLOPs
√	×	×	×	96.0	89.1	92.6	62.6	19.88	57.0
√	√	×	×	93.4	90.3	94.4 (↑ 1.8)	64.7	39.81	62.7
√	×	√	×	95.7	91.7	93.7 (↑ 1.1)	66.7	23.71	69.8
√	×	×	√	92.8	92.6	93.9 (↑ 1.3)	66.4	19.94	56.7
√	√	√	×	94.8	92.5	95.1 (↑ 2.5)	67.4	43.74	75.9
√	√	×	√	93.5	93.8	95.3 (↑ 2.7)	67.1	39.90	62.6
√	×	√	√	94.2	93.1	94.8 (↑ 2.2)	67.6	23.97	69.9
√	√	√	√	95.1	93.4	95.8 (↑ 3.2)	67.7	43.81	75.7

**Table 9:** Inference performance comparison of different ablation variants. √ indicates the module is adopted; × indicates the module is not adopted.

Baseline	ES-MoE	MSAF	DMIFI	Params (M)	GFLOPs	Latency (ms)	FPS	GPU Mem (GB)
√	×	×	×	19.88	57.0	19.42	51.5	0.646
√	√	×	×	39.81	62.7	24.23	41.3	0.919
√	×	√	×	23.71	69.8	20.23	49.4	0.712
√	×	×	√	19.94	56.7	19.00	52.6	0.648
√	√	√	×	43.74	75.9	25.04	39.9	0.985
√	√	×	√	39.90	62.6	23.81	42.0	0.921
√	×	√	√	23.97	69.9	19.81	50.5	0.715
√	√	√	√	43.81	75.7	24.62	40.6	0.989

## Data Availability

Data will be available upon request from the corresponding authors. The original data presented in the study are openly available in publicly accessible repositories. The DIOR dataset can be found on Google Drive at https://drive.google.com/drive/folders/1UdlgHk49iu6WpcJ5467iT-UqNPpx__CC (accessed on 10 May 2026); the NWPU-VHR-10 dataset is available on GitHub at https://github.com/Gaoshuaikun/NWPU-VHR-10 (accessed on 12 May 2026); and the RSOD dataset is available on GitHub at https://github.com/RSIA-LIESMARS-WHU/RSOD-Dataset- accessed on 15 May 2026.

## References

[1] Cheng G., Han J. (2016). A survey on object detection in optical remote sensing images. ISPRS J. Photogramm. Remote Sens..

[2] Zhu X.X., Tuia D., Mou L., Xia G.-S., Zhang L., Xu F., Fraundorfer F. (2017). Deep learning in remote sensing: A comprehensive review and list of resources. IEEE Geosci. Remote Sens. Mag..

[3] Liu S., Zou H., Huang Y., Cao X., He S., Li M., Zhang Y. (2023). ERF-RTMDet: An improved small object detection method in remote sensing images. Remote Sens..

[4] Wen L., Cheng Y., Fang Y., Li X. (2023). A comprehensive survey of oriented object detection in remote sensing images. Expert Syst. Appl..

[5] Wei X., Yuan M. (2023). Adversarial pan-sharpening attacks for object detection in remote sensing. Pattern Recognit..

[6] Teng Z., Duan Y., Liu Y., Zhang B., Fan J. (2021). Global to local: Clip-LSTM-based object detection from remote sensing images. IEEE Trans. Geosci. Remote Sens..

[7] Ma W., Li N., Zhu H., Jiao L., Tang X., Guo Y., Hou B. (2022). Feature split–merge–enhancement network for remote sensing object detection. IEEE Trans. Geosci. Remote Sens..

[8] Zhang C., Lam K.-M., Wang Q. (2023). CoF-Net: A progressive coarse-to-fine framework for object detection in remote-sensing imagery. IEEE Trans. Geosci. Remote Sens..

[9] Deng Z., Sun H., Zhou S., Zhao J., Lei L., Zou H. (2018). Multi-scale object detection in remote sensing imagery with convolutional neural networks. ISPRS J. Photogramm. Remote Sens..

[10] Zhang G., Lu S., Zhang W. (2019). CAD-Net: A context-aware detection network for objects in remote sensing imagery. IEEE Trans. Geosci. Remote Sens..

[11] Yang X., Yang J., Yan J., Zhang Y., Zhang T., Guo Z., Sun X., Fu K. Scrdet: Towards more robust detection for small, cluttered and rotated objects. Proceedings of the IEEE/CVF International Conference on Computer Vision.

[12] Wang P., Sun X., Diao W., Fu K. (2019). FMSSD: Feature-merged single-shot detection for multiscale objects in large-scale remote sensing imagery. IEEE Trans. Geosci. Remote Sens..

[13] Wang H., Shi J., Karimian H., Wang F., Javed F., Liu B., Shi S., Li Z., Tao Y. (2026). CitrusNet: A vision transformer-CNN approach for citrus detection from multi-source imagery with multi-scale feature integration. Comput. Electron. Agric..

[14] Li K., Cheng G., Bu S., You X. (2017). Rotation-insensitive and context-augmented object detection in remote sensing images. IEEE Trans. Geosci. Remote Sens..

[15] Ding J., Xue N., Xia G.-S., Bai X., Yang W., Yang M.Y., Belongie S., Luo J., Datcu M., Pelillo M. (2021). Object detection in aerial images: A large-scale benchmark and challenges. IEEE Trans. Pattern Anal. Mach. Intell..

[16] Xu X., Dong S., Xu T., Ding L., Wang J., Jiang P., Song L., Li J. (2023). Fusionrcnn: Lidar-camera fusion for two-stage 3d object detection. Remote Sens..

[17] Song K., Wen H., Xue X., Huang L., Ji Y., Yan Y. (2023). Modality registration and object search framework for UAV-based unregistered RGB-T image salient object detection. IEEE Trans. Geosci. Remote Sens..

[18] Wen S., Guo W., Liu Y., Wu R. (2022). Rotated object detection via scale-invariant mahalanobis distance in aerial images. IEEE Geosci. Remote Sens. Lett..

[19] Ren S., He K., Girshick R., Sun J. (2015). Faster r-cnn: Towards real-time object detection with region proposal networks. Adv. Neural Inf. Process. Syst..

[20] Cai Z., Vasconcelos N. Cascade r-cnn: Delving into high quality object detection. Proceedings of the IEEE Conference on Computer Vision and Pattern Recognition.

[21] Sun P., Zhang R., Jiang Y., Kong T., Xu C., Zhan W., Tomizuka M., Li L., Yuan Z., Wang C. Sparse r-cnn: End-to-end object detection with learnable proposals. Proceedings of the IEEE/CVF Conference on Computer Vision and Pattern Recognition.

[22] Redmon J., Divvala S., Girshick R., Farhadi A. You only look once: Unified, real-time object detection. Proceedings of the IEEE Conference on Computer Vision and Pattern Recognition.

[23] Wang C.-Y., Yeh I.-H., Mark Liao H.-Y. Yolov9: Learning what you want to learn using programmable gradient information. Proceedings of the European Conference on Computer Vision.

[24] Wang A., Chen H., Liu L., Chen K., Lin Z., Han J., Ding G. (2024). Yolov10: Real-time end-to-end object detection. Adv. Neural Inf. Process. Syst..

[25] Khanam R., Hussain M. (2024). Yolov11: An overview of the key architectural enhancements. arXiv.

[26] Wang H., Shi J., Karimian H., Liu F., Wang F. (2024). YOLOSAR-Lite: A lightweight framework for real-time ship detection in SAR imagery. Int. J. Digit. Earth.

[27] Sapkota R., Cheppally R.H., Sharda A., Karkee M. (2025). YOLO26: Key architectural enhancements and performance benchmarking for real-time object detection. arXiv.

[28] Liu W., Anguelov D., Erhan D., Szegedy C., Reed S., Fu C.-Y., Berg A.C. Ssd: Single shot multibox detector. Proceedings of the European Conference on Computer Vision.

[29] Li Z., Yang L., Zhou F. (2017). FSSD: Feature fusion single shot multibox detector. arXiv.

[30] Carion N., Massa F., Synnaeve G., Usunier N., Kirillov A., Zagoruyko S. End-to-end object detection with transformers. Proceedings of the European Conference on Computer Vision.

[31] Zhu X., Su W., Lu L., Li B., Wang X., Dai J. (2020). Deformable detr: Deformable transformers for end-to-end object detection. arXiv.

[32] Zhang H., Li F., Liu S., Zhang L., Su H., Zhu J., Ni L.M., Shum H.-Y. (2022). Dino: Detr with improved denoising anchor boxes for end-to-end object detection. arXiv.

[33] Li K., Wan G., Cheng G., Meng L., Han J. (2020). Object detection in optical remote sensing images: A survey and a new benchmark. ISPRS J. Photogramm. Remote Sens..

[34] Cheng G., Zhou P., Han J. (2016). Learning rotation-invariant convolutional neural networks for object detection in VHR optical remote sensing images. IEEE Trans. Geosci. Remote Sens..

[35] Long Y., Gong Y., Xiao Z., Liu Q. (2017). Accurate object localization in remote sensing images based on convolutional neural networks. IEEE Trans. Geosci. Remote Sens..

[36] Lin T.-Y., Maire M., Belongie S., Hays J., Perona P., Ramanan D., Dollár P., Zitnick C.L. Microsoft coco: Common objects in context. Proceedings of the European Conference on Computer Vision.

[37] Wang J., Yang W., Guo H., Zhang R., Xia G.-S. Tiny object detection in aerial images. Proceedings of the 2020 25th International Conference on Pattern Recognition (ICPR).

[38] Wu Y., Zhang K., Wang J., Wang Y., Wang Q., Li Q. (2020). CDD-Net: A context-driven detection network for multiclass object detection. IEEE Geosci. Remote Sens. Lett..

[39] Han W., Li J., Wang S., Wang Y., Yan J., Fan R., Zhang X., Wang L. (2022). A context-scale-aware detector and a new benchmark for remote sensing small weak object detection in unmanned aerial vehicle images. Int. J. Appl. Earth Obs. Geoinf..

[40] Deng C., Wang M., Liu L., Liu Y., Jiang Y. (2021). Extended feature pyramid network for small object detection. IEEE Trans. Multimed..

[41] Gao T., Xia S., Liu M., Zhang J., Chen T., Li Z. (2025). Msnet: Multi-scale network for object detection in remote sensing images. Pattern Recognit..

[42] Zhang J., Lv J., Zhang H., Li M., Huang M. (2026). MDADet: A Multimodal Dynamic Adaptation Framework for Efficient Small Object Detection in Aerial Images. IEEE Trans. Geosci. Remote Sens..

[43] Zhang Y., Liu T., Yu P., Wang S., Tao R. (2024). SFSANet: Multiscale Object Detection in Remote Sensing Image Based on Semantic Fusion and Scale Adaptability. IEEE Trans. Geosci. Remote Sens..

[44] Shi S., Fang Q., Xu X., Dong D. (2025). Multiscale Gaussian Attention Mechanism for Tiny Object Detection in Remote Sensing Images. IEEE Trans. Geosci. Remote Sens..

[45] Wu Q., Li Y., Yin J., You X. (2025). LGC-YOLO: Local-Global Feature Extraction and Coordination Network With Contextual Interaction for Remote Sensing Object Detection. IEEE J. Sel. Top. Appl. Earth Obs. Remote Sens..

[46] Lin T.-Y., Dollár P., Girshick R., He K., Hariharan B., Belongie S. Feature pyramid networks for object detection. Proceedings of the IEEE Conference on Computer Vision and Pattern Recognition.

[47] Ghiasi G., Lin T.-Y., Le Q.V. Nas-fpn: Learning scalable feature pyramid architecture for object detection. Proceedings of the IEEE/CVF Conference on Computer Vision and Pattern Recognition.

[48] Tan M., Pang R., Le Q.V. Efficientdet: Scalable and efficient object detection. Proceedings of the IEEE/CVF Conference on Computer Vision and Pattern Recognition.

[49] Liu S., Qi L., Qin H., Shi J., Jia J. Path aggregation network for instance segmentation. Proceedings of the IEEE Conference on Computer Vision and Pattern Recognition.

[50] Zhang T., Zhang X., Zhu P., Chen P., Tang X., Li C., Jiao L. (2021). Foreground refinement network for rotated object detection in remote sensing images. IEEE Trans. Geosci. Remote Sens..

[51] Zhao Y., Lv W., Xu S., Wei J., Wang G., Dang Q., Liu Y., Chen J. Detrs beat yolos on real-time object detection. Proceedings of the IEEE/CVF Conference on Computer Vision and Pattern Recognition.

[52] Huang S., Lu Z., Cun X., Yu Y., Zhou X., Shen X. Deim: Detr with improved matching for fast convergence. Proceedings of the Computer Vision and Pattern Recognition Conference.

[53] Zhao W., Deng X., Gao F., Liu C. (2025). Position-DETR: Step-by-step position-guided small object detection in remote sensing images. IEEE Trans. Geosci. Remote Sens..

[54] Zhi Y., Zhao J., Song C., Ma M., Mei S. (2025). SAFF-DETR: An End-to-End Object Detection Network for Remote Sensing Images With Targets of Varying Sizes Based on Scale Adaptation and Frequency Fusion. IEEE Trans. Geosci. Remote Sens..

[55] Li J., Shi Y., Hong Q., Jia Y. (2025). A Scale-Aware Multi-Domain DETR for Small Object Detection in UAV Remote Sensing Imagery. IEEE Trans. Geosci. Remote Sens..

[56] Lin X., Peng J., Gan Z., Zhu J., Liu J. (2025). YOLO-Master: MOE-Accelerated with Specialized Transformers for Enhanced Real-time Detection. arXiv.

[57] Jacobs R.A., Jordan M.I., Nowlan S.J., Hinton G.E. (1991). Adaptive mixtures of local experts. Neural Comput..

[58] Tian Z., Shen C., Chen H., He T. Fcos: Fully convolutional one-stage object detection. Proceedings of the IEEE/CVF International Conference on Computer Vision.

[59] Zhang P., Liu J., Zhang J., Liu Y., Shi J. (2025). HAF-YOLO: Dynamic feature aggregation network for object detection in remote-sensing images. Remote Sens..

[60] Yuan H., Zhang B., Wang Y., Qiang Q. (2026). From Structural Degradation to Semantic Misalignment: A Unified Frequency-Aware Compensation Framework for Remote Sensing Object Detection. Remote Sens..

[61] Yang F., Chen G., Duan J. (2024). Skip-Encoder and Skip-Decoder for Detection Transformer in Optical Remote Sensing. Remote Sens..

[62] Li Z., Wang Y., Xu D., Gao Y., Zhao T. (2025). TBNet: A texture and boundary-aware network for small weak object detection in remote-sensing imagery. Pattern Recognit..

[63] Li Y., Hou Q., Zheng Z., Cheng M.-M., Yang J., Li X. Large selective kernel network for remote sensing object detection. Proceedings of the IEEE/CVF International Conference on Computer Vision.

[64] Hu K., Li J., Ji N., Xiang X., Jiang K., Gao X. (2026). Knowledge distillation with spatial semantic enhancement for remote sensing object detection. ISPRS J. Photogramm. Remote Sens..

[65] Li J., Li H., Xu H., Song R., Li Y., Du Q. (2024). Background suppression network with attention collapse inhibited transformer for optical remote sensing object detection. IEEE Trans. Geosci. Remote Sens..

[66] Dong R., Xu D., Zhao J., Jiao L., An J. (2019). Sig-NMS-based faster R-CNN combining transfer learning for small target detection in VHR optical remote sensing imagery. IEEE Trans. Geosci. Remote Sens..

[67] Shi L., Kuang L., Xu X., Pan B., Shi Z. (2021). CANet: Centerness-aware network for object detection in remote sensing images. IEEE Trans. Geosci. Remote Sens..

[68] Ma J., Wang G., Yin R., He G., Zhou D., Long T., Adam E., Zhang Z. (2026). Wind Turbines Small Object Detection in Remote Sensing Images Based on CGA-YOLO: A Case Study in Shandong Province, China. Remote Sens..

[69] Gao T., Li Z., Wen Y., Chen T., Niu Q., Liu Z. (2023). Attention-free global multiscale fusion network for remote sensing object detection. IEEE Trans. Geosci. Remote Sens..

[70] Yuan Y., Wei Y., Zhou X., Guo Y., Chen J., Jiang T. (2025). YOLO-SBA: A multi-scale and complex background aware framework for remote sensing target detection. Remote Sens..

